# Bicycle-related traumatic injury hospitalizations: six years descriptive analysis in Qatar

**DOI:** 10.5249/jivr.v11i2.1162

**Published:** 2019-07

**Authors:** Husham Abdelrahman, Ayman El-Menyar, Brijesh Sathian, Rafael Consunji, Ismail Mahmood, Mohammed Ellabib, Hassan Al-Thani

**Affiliations:** ^*a*^Department of Surgery, Trauma Surgery, Hamad General Hospital, Doha, Qatar.; ^*b*^Department of Surgery, Trauma Surgery, Clinical Research, Hamad General Hospital, Doha, Qatar.; ^*c*^Clinical Medicine, Weill Cornell Medical School, Doha, Qatar.; ^*d*^Injury Prevention Program, Trauma Surgery Section, Hamad General Hospital, Doha, Qatar.

**Keywords:** Bicycle-related, Traumatic Injuries, Helmet, Risk factors, Qatar

## Abstract

**Background:**

Bicycle riding is a widely practiced mode of transportation, commuting, competition, fitness and recreation. We aimed to describe the incidence, risk factors and outcomes of Bicycle-Related Traumatic Injury (BRTI) in a Middle Eastern country.

**Methods:**

Data were extracted from a prospectively collected trauma registry over a period of six years (2010- 2015) from the national trauma center. Demographics and clinical characteristics of patients, and outcomes were analyzed.

**Results:**

There were 150 patients with a mean age of 27.2±16.6 years, 98% were males, 86.6% were hit by a car and 8.7% died. The average annual incidence of BRTIs was 1.3 per 100,000 populations. The mean Glasgow Coma Score (GCS) and injury severity score (ISS) were12.7±4.0 and 13.6±9.8; respectively. Almost one-third of cases had an ISS of 9-15. The most commonly injured region was the head (47%) followed by a lower extremity (30%), chest (25%), upper extremity (21.3%), spine (20.7%), abdomen (18.7%) and (7%) pelvis.

**Conclusions:**

BRTI is relatively uncommon in Qatar; however, it is characterized by a distinct epidemiology with a considerable mortality. Young male nationals, recreational cyclists and expatriate young commuter cyclists comprise the majority of victims and should be the focus of primary prevention efforts. Complementary prevention should aim at enforcing helmet laws to reduce fatal head injuries, and educating motorists of safer practices around cyclists.

## Introduction

Trauma is one of the leading causes of deaths worldwide especially among individuals under the age of forty and this age group remains as a top priority for prevention efforts.^1^

Traffic-related injuries are the most common mechanism of injury in Qatar and worldwide; these include motor vehicle crashes (MVCS), motorcycle crashes (MCC), pedestrian and bicycle-related traumatic injuries (BRTI).^2^ The latter is a very important cause of injury in many places in Europe, US, Southeast Asia and worldwide as cycling is a very popular means of recreation and transportation.^3^

Children are particularly at high risk; however, the incidence is rising among middle-aged and even elderly riders as well. It has noted to be a cheaper alternative to otherwise pricey transportation in addition to the physical fitness, exercise, and leisure.^4^

The likelihood to sustain multiple serious injuries in a bicycle crash is high considering the relatively exposed body regions of the cyclist. Mandatory bicycle helmet use legislation, based on the evidence of effectiveness in decreasing associated head injuries, is highly advocated;^5^ nevertheless the reported compliance is poor.^6^ Certain crash risk factors have been reported, including familiarity with bicycle type, crash mode, rule violations and age of the rider.^7^ Environmental factors include municipality bicycle roadway infrastructure planning, including proper separate bicycle paths.^8^ Perceived risk among road users is an interesting factor that influences the use of safety measures.^9^ Alcohol consumption is another risk factor,^10^ as well as increased cycling mileage and length of a time cycling.^11^ BRTIs are highly potentially preventable; both on a primary and secondary basis.^12^ Potential differences exist in bicycling-related risk and experience of injury by population subgroup; boys and new immigrants.^13,14^ Public roads over malls and bicycle tracks are important risky places.^15^ Despite all of this evidence, there is a lack of studies addressing this increasingly popular mode of transportation in the Arab Middle East region in general and in the state of Qatar.

We aimed to study the bicycle-related traumatic injuries that warranted admission to the level 1Hamad Trauma Center; the national trauma center of the state of Qatar, to define the incidence, risk factors, timing, type and patterns of injuries and outcomes of BRTIs in Qatar, in order to inform future focused injury prevention efforts.

## Patients and Methods

The primary sample for this study was collected from consecutive data, for all patients with BRTI, under ICD-9-CM Diagnosis Code E826 involving pedal cycle between January 1, 2010 and December 31, 2015. 

Data were obtained from the trauma registry of the Hamad Trauma Center [HTC], the national trauma referral center of Qatar. The HTC is the only Level I Trauma Center serving the entire country, receiving more than 98% of the county’s trauma patients. This trauma registry is compliant with both the National Trauma Data Bank [NTDB] and Trauma Quality Improvement Program [TQIP] of the American College of Surgeons-Committee on Trauma. 

Variables collected and analyzed included: demographic data (age, gender, nationality), mechanism of injury details (Cyclist hit by car, Fall from bicycle), timing of the injury, injury severity score (ISS), diagnosis, Glasgow Coma Scale (GCS), ICU admission, ventilator days, hospital length of stay (LOS), disposition, use of helmets, and mortality, National population data from the Ministry of Development Planning and Statistics was used to compute for population-based BRTI incidence rates [per 100,000 population].^8^

Severely injured BRTI patients who died at the scene before arriving to the hospital and those with mild injuries who were seen, treated and discharged from the Emergency Department or at Primary Health Care Centers (PHCCs) were not included in this study. 

**Statistical Analysis**

Descriptive analyses were reported as frequencies and percentages for categorical variables. Continuous variables’ central tendency was described using means with standard deviations for variables with normal distribution (as assessed by the Shapiro-Wilk test), and medians with ranges for variables with non-normal distribution. Categorical variables were compared using chi-square test or Z-test of proportions as appropriate. Also, 95% Confidence intervals were used wherever it is applicable. A 2-tailed P value<0.05 was considered statistically significant. BRTI characteristics are presented using frequency percentages for categorical variables, mean +/- standard deviation (SD) for continuous variables, line graphs, and bar graphs. Year wise cases were calculated by allotting the identified cases into their corresponding years. Then for the year wise incidence rate the total BRTI cases per year divided by the corresponding year population of Qatar and converted into 100,000. Data were analyzed using PASW statistics 18, SPSS Inc., USA.

Ethical approval for this study was obtained from Research Ethics Committee, Medical Research Center, Hamad Medical Corporation, Doha, Qatar [MRC# 16096/16].

## Results

**Overall data**

**Figure 1:**
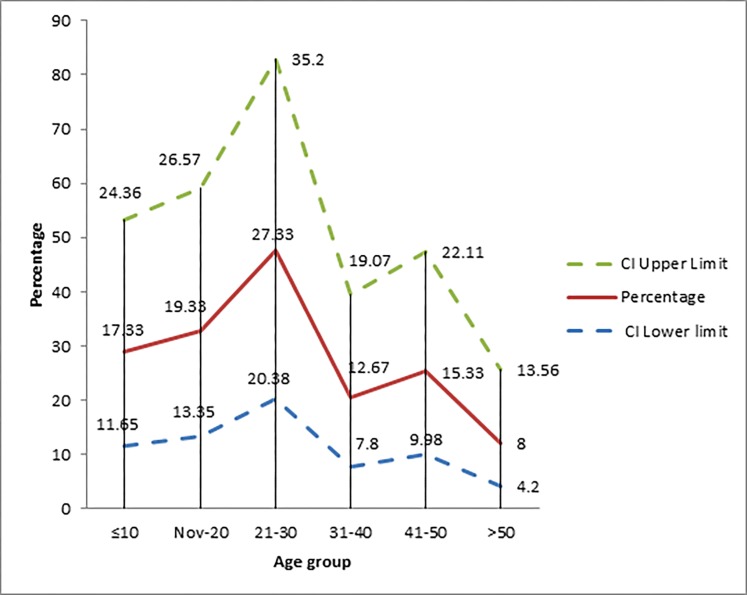
Bicycle injuries by age groups.

During the study period; 150 patients were admitted to the Hamad Trauma Center with BRTI. The majority were males (147, 98%) and under 30 years of age (62.7%). The mean age was 27.2±16.6 and the most common age groups were 21 to 30 years [26.9 %] and 11-20 years [19.3%] (Figure 1). The documented use of bicycle helmets was very low (3.3%). The most commonly injured nationalities were Nepalese (16.7%), followed by Qataris (16.0%) and Sri Lankans (13.3%).

**Mechanism of injury**

The predominant mechanism of injury was collision with a car (MVC) (86.6%) followed by fall from a cycle (13.4%). Likewise, younger victims, less than 20 years, were more likely to be injured in a fall rather than a collision with a vehicle.

**Injury severity and admission**

The mean GCS was 12.7±4.0. The mean head AIS was 3.6±1.5 and chest AIS was 3.1±1.1. One-fourth [25.3%] of patients needed admission to the Trauma Intensive Care Unit (TICU) and 5 [3.3%] patients with severe head injury died in the ED but the majority [55.3%] were admitted to the Trauma Surgical Unit for routine care. The mean ICU length of stay was 4 days (1-49) and the mean ventilator days was 5 (1-20). The overall mean hospital length of stay was 5 days (1-148) and the overall mortality rate was 8.7% (n=13) (Table 1).

**Table 1:** Frequency, pattern and outcomes of bicycle-related injuries.

Males (n, %)	147 (98.0%)
Age (mean ±SD; years)	27.2±16.6
Crash type (n, %)	
Cyclist hit by car	129 (86.6%)
Fall from bicycle	20 (13.4%)
Documented Helmet use	4 (3.3%)
Glasgow coma score initial (mean ±SD)	12.7±4.0
Head AIS (mean ±SD)	3.6±1.5
Upper extremity AIS (mean ±SD)	2.0±0.2
Lower extremity AIS (mean ±SD)	2.7±1.1
Chest AIS (mean ±SD)	3.1±1.1
Spine AIS (mean ±SD)	2.3±0.6
Abdomen AIS (mean ±SD)	2.8±1.4
Pelvis AIS (mean ±SD)	2.5±0.9
Injury Severity Score (ISS) (mean ±SD)	13.6±9.8
Mild (ISS≤8)	44 (30.3%)
Moderate (ISS 9-15)	56 (38.6%)
Severe (ISS≥16)	45 (31.0%)
Emergency department disposition (n, %)	
Operating room	24 (16.0%)
Intensive care unit	38 (25.3%)
Trauma Floor	83 (55.3%)
Died	5 (3.3%)
Hospital discharge Disposition	
Home	114(76%)
Transfer	23 (15.6%)
In-hospital death	8 (5.3%)
Intensive Care Unit length of stay (n, Median, range, days)	38, 4 (1-49)
Ventilator days (n, Median, range, days)	41, 5 (1-20)
Hospital length of Stay (Median, range, days)	5 (1-148)
Overall mortality (n, %)	13 (8.7%)

The mean ISS, for all patients, was 13.6±9.8 with a nearly equal distribution of injuries, for mild and severe ISS with most classified as moderate, ISS 9-15 [38.6%]. Most severe injuries [55.5%] were sustained by young adults, ages 21-40 years while most injuries to younger patients and children were mild to moderate (Table 2). 

**Table 2:** Bicycle-related injuries, by ISS & age-group, both sexes, (2010-2015) (n=150) Trauma Registry, Hamad Trauma Center, Doha, Qatar.

Severity [ISS]				
AGE GROUP	N	Mild n[%]	Moderaten[%]	Severen[%]
0-10	25	12 [48]	10 [40]	3 [12]
11-20	28	8 [28.5]	13 [46.5]	7 [25]
21-30	39	9 [23]	15 [38.5]	15 [38.5]
31-40	18	6[33.5]	2 [11]	10 [55.5]
41-50	23	7 [30.5]	10 [43.5]	6 [26]
50-100	12	2 [17]	6 [50]	4 [33]
**Day**				
Week day	79	23 [29]	32 [40.5]	24 [30.5]
weekend	66	21 [32]	24 [36]	21 [32]
**HOURS**				
1-3	5	1 [20]	1 [20]	3 [60]
4-8	10	1 [10]	3 [30]	6 [60]
9-12	7	3 [43]	2 [28.5]	2 [28.5]
13-16	15	3 [20]	8 [53]	4 [27]
17-20	34	10 [30]	12 [37]	11 [33]
21-24	20	7 [35]	6 [30]	7 [35]

**Injury location**

The most commonly injured region was the head (46.7%) followed by lower extremity (30%), chest (24.7%), upper extremity (21.3%), spine (20.7%), abdomen (18.7 %), and pelvis (7.3%). When patients with head injury were classified by age-group, children below 10 years had the lowest mean head AIS [2.63], and those aged 31-40 had the highest mean head AIS [4.44] ( Table 3). 

**Table 3:** Age Group and Head AIS.

Age group	Number	Mean ± SD	Median (Range)
0-10	11	2.6±1.1	3 (1, 5)
11-20	15	3.3±0.9	3 (2, 5)
21-30	22	3.9±1.9	3 (2, 9)
31-40	9	4.4±1.9	4 (3, 9)
41-50	7	3.7±1.3	3 (2, 5)
>50	6	3.0±0.6	3 (2, 4)

**Annual incidence and timing of admission**

**Figure 2:**
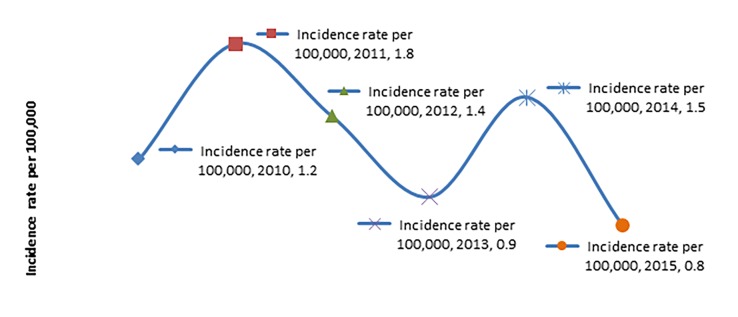
Incidence rate per 100,000, Bicycle-related injuries, by year, both sexes, all ages, (2010-2015) (n=150) Trauma Registry, Hamad Trauma Center, Doha, Qatar and Qatar Statistics Authority.

The highest reported incidence of BRTI was in the year 2014 and the year 2011(1.8 per 100,000 and 1.5 per 100,000 respectively). The average incidence of BRTI per 100,000 Qatar inhabitants was 1.3 per 100,000 during the study period (Figure 2). 

**Figure 3:**
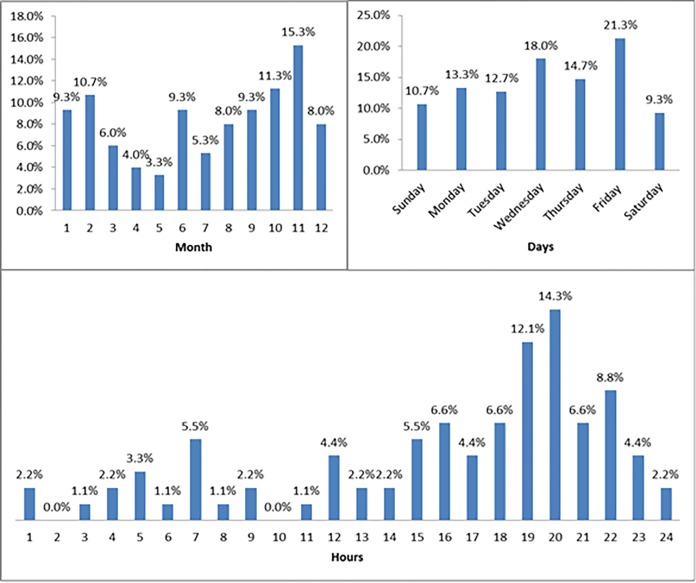
Bicycle-related injuries, by month of injury, days, and hours.

Most BRTIs [43.3%] were admitted during the coolest months, November to February, with the least BRTIs [25.9%] during the warmest months, May to August (Figure 3).

The majority of BRTIs [69.4%] occurred on weekdays, Sunday to Wednesday, but the peak daily incidence was on Friday [21.3%]. There was a statistically insignificant trend towards more moderate and less mild injuries on weekdays when compared to weekends. 

By age-group, there were more BRTIs on weekdays for the working age groups, ages 21-50 (60%) while those less than 20 years old were more often injured on weekends (55%).

Most BRTIs [37.2%] occurred during the late afternoon and early commuting times, from 1700-2000 H, with the least [5.5%] during the early morning period, 00:00-03:00H. The peak hourly incidence [14.3%] was from 20:00-21:00H. From 00:00-08:00 were the hours with more severe BRTIs, the majority of BRTIs in these periods had ISS≥16, when compared to other time periods.

## Discussion

The current study describes the burden of BRTI related hospitalizations in the state of Qatar along with the type and location of injury. One out of every 67 patients admitted to the national trauma center has a BRTI. They almost uniformly affect males, particularly those who are 21-30 years old and the majority has been involved in collisions with cars. The teens represent the riskiest group considering their cycling behavior and recklessness.The incidence of BRTIs on weekends is more than twice that on week days.

HTC receives around 1500 to 2000 admission per year, 60% of these admissions are traffic related and are related to vehicle users and pedestrians. The incidence of bicycle related injury in Qatar is not known and never reported before. This center was established at end of 2007 and we wanted to get a complete set of data so we allowed a maturation period of 3 years for the registry. This report addresses the epidemiology and outcomes of BRTIs by age group, nationality, temporal pattern, mechanism and anatomic location of injury, injury severity and outcome, using data from a nationally representative and multi-year sample from the national trauma referral hospital. The results are reflective of all moderate to severe BRTIs, serious enough to merit admission at the Trauma Center.

The incidence of BRTI in Qatar is low compared to the prior reports such as UAE, USA, Europe, Iran, Hong Kong, India which all reported an increase in BRTIs.^16-22^ Also the influence of age on the mode of injury and type of sustained injuries are of interest to define a particular high risk group of the community.

In the current study, the two most commonly involved nationalities were from the South Asian subcontinent, namely Nepalese and Sri Lankan that represents a majority in the population census dominant nationalities in Qatar. Furthermore the majority of male workers use bicycle for commuting and thus represent a high risk group; while the national Qataris tend to be younger and use it mainly for exercise.

Weekends, especially Fridays, the cooler months (November to February), and the early evening, from 17:00-20:00 H, showed the peak incidence for BRTIs and this define high risk times for this important mode of injury. The average annual incidence rate was 1.0 per 100,000, and one in 11 (9%) succumbed to his injuries.

As far as we know, this is the first report of BRTIs in Qatar and the second one from the Arab Middle East region. That’s why we need to compare the two regional studies. This study could be an addition and an update to the research on BRTIs that has been conducted in the region; Hefny et al described 130 hospitalized BRTIs from 2001-2007 in UAE.^22^ The 2 studies from Qatar and UAE have commonalities in the male predominance, very low helmet utilization, and expatriate majority of BRTIs. The hospitalization rates for BRTI in the Al-Ain (5.9 per 100,000) were four times higher than in Qatar (1.3 per 100,000). Temporal patterns were different for the time of day, 00:00H vs 20:00H peak incidence, but similar for peak day and month incidence. Leading mechanisms of injury were the same, collision with vehicle and falls, and also the most common anatomical locations of injuries were head and extremities. There were differences between the 2 studies regarding the severity of injuries as measured using arrival GCS (15 vs. 12.7), and median/mean ISS (4 vs. 13.6). This could explain the doubling of the rate for ICU admission and the 6-fold higher mortality rate in the present report.

Most BRTIs in Qatar affect working age males in the cooler months similar to Al-Ain in the UAE. In contrast, many other studies from the rest of the world reported that the bicycle injuries were more common among male children and adolescents in the summer months, and by single-bicycle events.^11-15,17,18^

The disparity in findings can be partially explained by the unique demographics of Qatar and the GCC region, where 65% of the general population are males between the ages of 20-65 years as most of them are expatriate workers. Also, the hot climate, with a day-time temperature of 40oc degree and above, could limit the outdoor activities and explain the lower incidence of BRTIs in Qatar between May and October.

Of note, the majority of Qatari nationals affected by BRTIs were males, younger than 20 years old, a finding that is similar to prior data from the UAE and more in line with results from other more homogenous populations.^9,11-14,16,19,20,22^

Yeung et al reported that the young cyclist riding behavior influences the injury pattern.^21^ Other authors studied^22,23^ the temporal distribution and reported higher incidence of BRTIs at night, weekends; and during January and July in contrast to October and February in our study. Yeung et al also reported head injury as second to lower extremities in their cohort with a mortality rate of 1.5%.^21^ Juhra et al^24^ reported head injury rate 26% in a study from Germany as a leading cause of admission.

The higher incidence for evening related collisions as well as hit by other vehicles can be explained by the practice of driving on the main roads and against the traffic stream , the lower visibility during night and the lower percentage in using protective measures like helmet, reflective lights, and gears (shoes, gloves, knee pads, jacket and padded shorts).^12^ The lack of helmet use may have contributed to the high incidence of head injuries in our cohort compared to other reports.^6,16,22^ However low helmet use compliance seems to represent a global problem; in a report on BRTI from Germany the use of helmet was only in 6.4% and in another report from Hong Kong it was only 0.4%.^21,24^ There is an urgent need to reinforce legislation on the mandatory use of helmet for all riders as well as the provision of separate tracks for cyclist and bicycle related preventive measures like high visibility lights. ^25^

In the US, with a very different demographic and meteorological profile than Qatar and UAE, Haileyesus et al^26^ reported that most of BRTIs occurred during ‘warmer’ months, from May to September. The national hospitalization rate for BRTIs in the US, from 2001-2004, was reported as 2.7 per 100,000^17^ which is more than double the average incidence rate we documented in Qatar during the study period. While there is no data that document cyclist exposure to BRTI risk, like bicycle usage rates in terms of vehicle km traveled yearly, it is postulated that weather and lack of a ‘cycling’ culture contribute to the markedly lower BRTI rate in Qatar. We extrapolate our data to the national US data, as reported by Haileyesus et al, and then our patients represent roughly 12.5% of all BRTIs annually.^26^

Head was the most commonly injured region and contributes to poor outcome. Despite the existence of law that requires all cyclists to wear a helmet, since 2007, awareness of its existence and enforcement are still low [Traffic Law, Article 48 of Law no. 19 of 2007].^27^ The effect of the low usage rate of helmets, in this study population, can clearly be seen in the high mean head AIS of the 5 deaths in the ED; similarly all in-hospital deaths occurred in patients with brain injury. As described in a recent systematic review, head injuries might be the reason for the majority of mortality in BRTIs and this can be addressed by increasing helmet use that could reduce the risk of brain injury by 88%.^28^ Helmet use legislation based on evidence of effectiveness in decreasing associated head injuries is highly advocated; nevertheless, the compliance remains unsatisfactory in the developing countries.^6,15^

The vulnerability of cyclists is manifested in their higher likelihood to sustain multiple serious injuries especially considering their relatively exposed body, and the energy transfers that takes place from the initial impact to the numerous secondary energy transfers between the cyclist and the colliding vehicle, fixed objects and the ground. In this study, fractures of the extremities as well as chest, spine and abdomen were also common as was the polytrauma nature of BRTIs.

If we compare the anatomical location of injury in our study, with that from other studies, extremities (lower and upper together) were less common but head (46.7%), abdominal (18.7%), thoracic (24.7%) injuries were more common.^21,24^

Certain risk factors have been identified including familiarity of bicycle’s type, crash mode and rule violations, age of the rider; and municipality bicycle roadway infrastructure (i.e., proper separate bicycle paths).^4,7^ It has been noted that males and new migrants are at more risk of BRTIs.^11,13^

BRTIs are highly preventable; both on a primary and a secondary basis.^12^ Applying local data, from our study, to the Haddon Phase-Factor Matrix a well-known epidemiologic tool allows us to identify high-risk groups and possible areas for future research. It also allowed us to identify targets for both primary [high-visibility bike and rider, designated bike lane and well-lit roads], and secondary [enforcement of helmet law, increased affordability and awareness of helmet safety advantage], preventive programs for BRTIs in Qatar (Table 4).

**Table 4:** Haddon’s Matrix for DBRTI.

	Human	Environment		
		Agent/Carrier	Social	Physical
Pre-event	Qatari, Expatriate, Age, Sex, Nature of use [commute, commerce, recreation] Visibility [clothing], Co-morbid conditions, Fatigue, Alcohol, Substance/s, Medications, experience,	Age/Condition of Bike [brakes, tires] Visibility of bike-reflectors, Size of bike/rider	Bike safety awareness, soci-economic status- affordability of helmet, biking ‘culture’, assault risk?	Temperature, lighting, time of day,visibility – sandstorm, dust, fog, designated bike lane, mixing of road user types, traffic flow, volume and speed, road surface
Event	Protective gear [helmet, clothing]		Helmet laws,attitude to cyclists, incentives for helmet use?	traffic flow, volume and speed, road type- rural/urban
Post-event	Biking alone, Mobile phone, Self-care – 1st Aid		EMS, Rehab, Trauma Center	traffic flow, volume and speed [re-injury], road type- rural/urban

The unavailability of more precise measures of exposure of cyclists in Qatar, like bicycle km traveled or annual time of bicycle riding/use, ‘forces’ the authors to use population counts as an exposure measure when measuring risks. 

This study shares limitations that are inherent to many retrospective analyses that utilize clinical patient registry data to inform primary prevention initiatives: incomplete data entry with respect to pre-event circumstances, use or non-use of protective tools , crash characteristics [i.e. colliding vehicle/s and/or fixed object/s], patient co-morbid conditions and nature of transport [i.e. for commuting, recreation or commerce]. Furthermore a lacking piece of relevant details is the deficiency in focusing on characterizing the ‘victim’ or affected individual in bicycle related research and registry databases. Given that a cyclist is one of the most vulnerable road users, it would be more informative if a more thorough analysis is conducted of each crash that results in a BRTI because this would describe the physical environment of the crash or fall, characterize the ‘offending’ driver or fixed object that collides with the cyclist and even evaluate the roadworthiness of the bicycle itself. A complete description of these contributory factors will identify more ‘low hanging fruit’ as the foci of future BRTI prevention programs. Moreover, this report may under-report BRTIs in the country because it does not describe the mild or less severe BRTIs that did not need trauma center admission during the study period; which should be the focus of future research.

The wide diversity and temporal variability of Qatar’s population as well as the discrepancies in the nature of bicycle use, commuting versus recreation, income levels [helmet affordability] and access to other means of transport should all be considered in future research on BRTIs.

## Conclusion

BRTI are rare in Qatar yet they are an important cause of death and morbidity with a unique epidemiology. Young male Qatari recreational riders and expatriate commuter cyclists are the risk groups that need special attention. Improving the road environment for cyclists i.e. designated and well-lit lanes, and encouraging the use of conspicuity enhancement methods for cyclists and bikes, i.e. reflectorized bikes and brightly colored cycling gear, are recommended for primary prevention of bike crashes. Increasing awareness of the safety advantage of bike helmets and the existent mandatory bike helmet laws and consistent enforcement of these laws should be the initial steps in promoting secondary prevention of BRTIs. 

**Acknowledgement**

The authors thank the entire registry database team in the Trauma Surgery Section, Hamad General Hospital, Doha, Qatar.

## References

[1] Ballestas T, Wilkinson C, Weeramanthri T (2011). Rise in bicycle-related injury hospitalisation rates in middle-aged adults, 2000-09. Aust N Z J Public Health.

[2] Ekman R, Welander G, Svanström L, Schelp L, Santesson P (2001). Bicycle-related injuries among the elderly--a new epidemic?. Public Health.

[3] Depreitere B, Van Lierde C, Maene S, Plets C, Vander Sloten J, Van Audekercke R, Van der Perre G, et al. Bicycle-related head injury: a study of 86 cases. Accid Anal Prev. 2004 Jul;36(4):561-7. 10.1016/S0001-4575(03)00062-9 15094408

[4] Schwartz HJ, Brison RJ. Bicycle-related injuries in children: a study in two Ontario emergency departments, 1994. Chronic Dis Can. 1996 Spring;17(2):56-62. 9079352

[5] Finvers KA, Strother RT (1996). Mohtadi. The effect of bicycling helmets in preventing significant bicycle-related injuries in children. Clin J Sport Med.

[6] Weiss BD (1994). Bicycle-related head injuries. Clin Sports Med.

[7] Hu F, Lv D, Zhu J, Fang J (2014). Related risk factors for injury severity of e-bike and bicycle crashes in Hefei. Traffic Inj Prev.

[8] Kerr ZY, Rodriguez DA, Evenson KR, Aytur SA (2013). Pedestrian and bicycle plans and the incidence of crash-related injuries. Accid Anal Prev.

[9] Fullerton L, Becker T (1991). Moving targets: bicycle-related injuries and helmet use among university students. J Am Coll Health.

[10] Spaite DW, Criss EA, Weist DJ, Valenzuela TD, Judkins D, Meislin HW (1995). A prospective investigation of the impact of alcohol consumption on helmet use, injury severity, medical resource utilization, and health care costs in bicycle-related trauma. J Trauma.

[11] Sgaglione NA, Suljaga-Petchel K, Frankel VH (1982). Bicycle-related accidents and injuries in a population of urban cyclists. Bull Hosp Jt Dis Orthop Inst.

[12] Cevik M, Boleken ME, Sogut O, Gökdemir MT, Karakas E (2013). Abdominal injuries related to bicycle accidents in children. Pediatr Surg Int.

[13] Davison CM, Torunian M, Walsh P, Thompson W, McFaull S, Pickett W (2013). Bicycle helmet use and bicycling-related injury among young Canadians: an equity analysis. Int J Equity Health.

[14] Nakayama DK, Pasieka KB, Gardner MJ (1990). How bicycle-related injuries change bicycling practices in children. Am J Dis Child.

[15] Eilert-Petersson E, Schelp L (1997). An epidemiological study of bicycle-related injuries. Accid Anal Prev.

[16] Eid HO, Bashir MM, Muhammed OQ, Abu-Zidan FM (2007). Bicycle-related injuries: a prospective study of 200 patients. Singapore Med J.

[17] Hamann C, Peek-Asa C, Lynch CF, Ramirez M, Torner J (2013). Burden of hospitalizations for bicycling injuries by motor vehicle involvement: United States, 2002 to 2009. J Trauma Acute Care Surg.

[18] Scuffham P, Alsop J, Cryer C, Langley JD (2000). Head injuries to bicyclists and the New Zealand bicycle helmet law. Accid Anal Prev.

[19] Karbakhsh-Davari M, Khaji A, Salimi J (2008). Bicycle-related injuries in Tehran. Arch Iran Med.

[20] Munivenkatappa A, Devi BI, Gregor TI, Bhat DI, Kumarsamy AD, Shukla DP (2013). Bicycle accident-related head injuries in India. J Neurosci Rural Pract.

[21] Yeung JH, Leung CS, Poon WS, Cheung NK, Graham CA, Rainer TH (2009). Bicycle related injuries presenting to a trauma centre in Hong Kong. Injury.

[22] Hefny AF, Eid HO, Grivna M, Abu-Zidan FM (2012). Bicycle-related injuries requiring hospitalization in the United Arab Emirates. Injury.

[23] Begg DJ, Langley JD, Chalmers DJ (1991). Bicycle road crashes during the fourteenth and fifteenth years of life. N Z Med J.

[24] Juhra C, Wieskötter B, Chu K, Trost L, Weiss U, Messerschmidt M (2012). Bicycle accidents - do we only see the tip of the iceberg? A prospective multi-centre study in a large German city combining medical and police data. Injury.

[25] Macpherson A, Spinks A (2008). Bicycle helmet legislation for the uptake of helmet use and prevention of head injuries. Cochrane Database Syst Rev.

[26] Haileyesus T, Annest JL, Dellinger AM (2007). Cyclists injured while sharing the road with motor vehicles. Inj Prev.

[27] Qatar Legal Portal (Al-Meezan). Law no. (19) of 2007 Regarding the Traffic Law. 2007, http://www.almeezan.qa/LawView.aspx?opt&LawID=3993&language=en, accessed 5 December 2017.

[28] Olivier J, Creighton P (2017). Bicycle injuries and helmet use: a systematic review and meta-analysis. Int J Epidemiol.

